# Sex-specific association of *SH2B3* and *SMARCA4* polymorphisms with coronary artery disease susceptibility

**DOI:** 10.18632/oncotarget.19720

**Published:** 2017-07-31

**Authors:** Yuqiang Ji, Yanbin Song, Qingwen Wang, Pengcheng Xu, Zhao Zhao, Xia Li, Nan Wang, Tianbo Jin, Chao Chen

**Affiliations:** ^1^ Key Laboratory of Resource Biology and Biotechnology in Western China, Northwest University, Ministry of Education, Xi’an, Shaanxi 710069, China; ^2^ School of Life Sciences, Northwest University, Xi’an, Shaanxi 710069, China; ^3^ Department of Cardiovascular Medicine, First Hospital of Xi’an, Xi’an 710002, China; ^4^ Department of Cardiovascular Medicine, Affiliated Hospital Yan’an University, Yan’an 716000, China; ^5^ Department of Hand Surgery, Hebei Province Cangzhou Hospital of Integrated Traditional and Western Medicine, Cangzhou, Hebei 061001, China

**Keywords:** coronary artery disease, *SH2B3*, *SMARCA4*, single nucleotide polymorphism, gene

## Abstract

To determine whether sex differences affect the association between genetic polymorphisms and coronary artery disease (CAD) in the Chinese Han population, we conducted a study comparing the frequency of *SH2B3* and *SMARCA4* variants in 456 CAD patients (291 men, 165 women) and 685 age-matched controls (385 men, 300 women). Ten single nucleotide polymorphisms (SNPs) in *SH2B3* and *SMARCA4* were genotyped using MassARRAY technology. Allelic and genotypic models and haplotype frequencies were compared between groups. Logistic regression was used to estimate the CAD risk associated with the genotypes. We found that the “A” alleles in both rs11879293 and rs12232780 of *SMARCA4* were associated with CAD risk in men (*p* = 0.036 and *p* = 0.001, respectively). The genetic model showed that *SH2B3* was associated with CAD susceptibility in both women and men, while *SMARCA4* was associated with reduced odds of CAD in men. *SH2B3* haplotypes were associated with decreased CAD risk in women (*p* = 0.007) and increased CAD risk in men (*p* = 0.047). By providing evidence for the sex-related association between *SH2B3* and *SMARCA4* gene variants and CAD susceptibility in the Chinese Han population, this study may help define useful diagnostic and preventive markers for these patients.

## INTRODUCTION

Coronary artery disease (CAD) is the most common form of heart disease. As a major cause of morbidity, mortality, and disability worldwide, it imposes a tremendous social and economic burden on society [1]. CAD is characterized by atherosclerosis, a process of cumulative deposition of low-density lipoprotein (LDL) cholesterol in the arteries supplying blood to the heart that eventually leads to impaired or absent blood supply and myocardial infarction [2]. CAD is a complex, multifactorial disease. Among its risk factors are smoking, advanced age, diabetes mellitus, high blood pressure, high-fat diet, obesity, infectious agents, increased total and LDL cholesterol in plasma, increased plasma triglycerides, and decreased plasma high-density lipoprotein (HDL) cholesterol [3, 4]. In recent years, considerable effort has been devoted to identifying genes and inherited DNA sequence variants that contribute to CAD risk [5, 6]. In addition, gender differences in the incidence of CAD have caught the interest of many clinical researchers. In the current study, we focused on several CAD-related genes: *CDKN2B-AS1*, *CYP1A1*, *PSRC1*, *APOC1*, *SH2B3*, and *SMARCA4*, previously addressed through genome-wide association studies (GWAS) and large-scale replication studies [7–9].

The *SH2B3* gene, located on chromosome 12 (12q24), is a member of the SH2B adaptor family. The SH2B3 protein has been associated with negative regulation of cytokine signaling. However, the specific functions of *SH2B3* remain unclear. Variants in this region have been shown to be associated with various other traits including blood pressure [10, 11], blood lipids [12], platelet count [13], and type-1 diabetes mellitus [14]. Correlations between the *SH2B3* gene and CAD susceptibility have been addressed by GWAS [15–17]. The *SMARCA4* gene is located on chromosome 19 (19q13) and is a member of the SWI/SNF family of proteins. Recent reports suggested that mutations in this gene cause small cell carcinoma of the ovary of hypercalcemic type [18], lung cancer [19], and other diseases [20, 21]. Combining GWAS data and case-control studies, we reported the association of *SMARCA4* with CAD risk [22–24]. To date, however, no studies investigated the possible correlations between *SH2B3* and *SMARCA4* genes and CAD susceptibility in the Chinese Han population.

To test the hypothesis that *SH2B3* and *SMARCA4* gene polymorphisms contribute differentially to coronary vascular pathology in men and women, we conducted a case-control study to examine the associations between single nucleotide polymorphisms (SNPs) in those genes and CAD risk in Chinese Han individuals.

## RESULTS

The demographic characteristics of the study population, including gender and age, are summarized in Table 1. The study included 456 CAD cases (291 males and 165 females) and 685 controls (385 males and 300 females). The mean age of males and females in the case group was 59.56 ± 12.18 years and 64.01 ± 10.74 years, respectively. In the control group, the mean age of males and females was 47.55 ± 10.66 years and 49.93 ± 7.74 years, respectively. There was a significant difference between genders’ ages in the case group (*p* = 0.000), but not in the control group (*p* = 0.063).

**Table 1:** Characteristics of the subjects

Characteristics	Cases (n = 456)		*p*	Controls (n = 685)		*p*
	Male	Female		Male	Female	
Count	291	165		385	300	
Age (mean ± SD years)	59.56±12.18	64.01±10.74	0.00*	47.55±10.66	49.93±7.74	0.063

*p < 0.05 indicates statistical significance

Basic information of candidate SNPs, such as chromosomal position, gene, allele, HWE test results, as well as minor allele frequency (MAF) by gender are shown in Table 2. Rs11879293 and rs12232780 in *SMARCA4* were associated with CAD risk (*p* = 0.036 and *p* = 0.001, respectively) in the male population only. In this group, rs2072382 was also significantly associated with CAD but was excluded due to significant deviation from HWE (*p* = 0.03). Meanwhile, an almost significant association was observed between *SH2B3* and *SMARCA4* genes and CAD risk in the women population.

**Table 2:** Sex-specific association of individual SNPs with CAD

SNP	Chr	Allele A^#^/B	Gene	Minor allele frequency, %													
				Men									Women				
				HWE	Cases	Controls	OR	95%	CI	*p*	HWE	Case	Control	OR	95%	CI	*p*
rs3742003	12	G/A	SH2B3	0.75	0.12	0.09	1.36	0.95	1.95	0.090	1.00	0.09	0.09	0.97	0.61	1.55	0.903
rs12580300	12	A/G	SH2B3	0.46	0.41	0.42	0.98	0.78	1.22	0.831	0.72	0.37	0.42	0.81	0.61	1.07	0.134
rs7309325	12	G/T	SH2B3	0.50	0.11	0.08	1.38	0.96	1.98	0.082	0.15	0.09	0.09	0.98	0.61	1.59	0.946
rs2078863	12	T/C	SH2B3	0.76	0.53	0.50	1.11	0.90	1.38	0.324	0.91	0.55	0.49	1.25	0.95	1.63	0.107
rs7296313	12	T/C	SH2B3	0.34	0.11	0.09	1.29	0.90	1.85	0.172	0.49	0.09	0.09	0.99	0.62	1.58	0.969
rs11879293	19	A/G	SMARCA4	0.70	0.22	0.27	0.76	0.59	0.98	0.036*	0.17	0.27	0.26	1.08	0.79	1.46	0.634
rs12232780	19	A/G	SMARCA4	0.78	0.18	0.25	0.64	0.49	0.84	0.001*	0.14	0.23	0.19	1.23	0.88	1.70	0.220
rs2072382	19	T/C	SMARCA4	0.03**^ψ^**	0.34	0.28	1.30	1.03	1.65	0.026*	0.19	0.31	0.28	1.15	0.86	1.55	0.343
rs1529729	19	C/T	SMARCA4	0.66	0.22	0.22	0.98	0.76	1.28	0.904	0.14	0.22	0.23	0.93	0.67	1.28	0.638
rs1122608	19	T/G	SMARCA4	0.41	0.08	0.10	0.74	0.51	1.08	0.120	1.00	0.09	0.08	1.22	0.76	1.96	0.411

SNP = single nucleotide polymorphism; CAD = coronary artery disease; OR = odds ratio;

95% CI = 95% confidence interval; Chr = chromosome; HWE = Hardy–Weinberg equilibrium

^#^Minor allele

*p* values were calculated from two-sided chi-square tests for either allele frequency

^ψ^Site with HWE P < 0.05 is excluded in the men population

* *p* < 0.05 indicates statistical significance

The association between each SNP and CAD risk was further assessed using unconditional logistic regression analysis including five genetic models: codominant, dominant, recessive, overdominant, and additive (Table 3). The minor allele “A” of rs12580300 in *SH2B3* was associated with CAD risk in the female population under the codominant (*p* = 0.043), dominant (AA + AG vs. GG: OR = 0.59, 95% CI: 0.35-0.97; *p* = 0.039), and additive (OR = 0.63, 95% CI: 0.44-0.91; *p* = 0.012) models. In this same population, the allele “T” in *SH2B3* rs2078863 was associated with decreased odds of CAD risk in the codominant (*p* = 0.022), recessive (CC vs. TT + TC: OR = 0.44, 95% CI: 0.24-0.82; *p* = 0.007), and additive (OR = 0.64, 95% CI: 0.44-0.91; *p* = 0.012) models. Interestingly, in the *SH2B3* gene, rs3742003 in the codominant and overdominant models, and rs7309325 in the overdominant model, were associated with increased CAD risk in the male population. Meanwhile, the *SMARCA4* SNPs rs11879293 in the codominant, dominant, and additive models, and rs12232780 in the co-dominant, dominant, recessive, overdominant, and additive models, were associated with decreased CAD risk in this population

**Table 3:** Genotypes of *SH2B3* and *SMARCA4* polymorphisms and CAD risk associations (age-adjusted)

					Men			Women		
SNP	Model	Genotype	Control	Case	OR (95% CI)	*p*	Control	Case	OR (95% CI)	*p*
rs3742003	Codominant	A/A	319 (83.1%)	224 (77%)	1	0.076	246 (82%)	136 (82.4%)	1	0.960
		G/A	63 (16.4%)	67 (23%)	1.57 (1.01-2.42)		52 (17.3%)	28 (17%)	0.99 (0.53-1.85)	
		G/G	2 (0.5%)	0 (0%)	0.00 (0.00-NA)		2 (0.7%)	1 (0.6%)	0.59 (0.02-20.73)	
	Dominant	A/A	319 (83.1%)	224 (77%)	1	0.054	246 (82%)	136 (82.4%)	1	0.950
		G/A-G/G	65 (16.9%)	67 (23%)	1.53 (0.99-2.36)		54 (18%)	29 (17.6%)	0.98 (0.53-1.81)	
	Recessive	A/A-G/A	382 (99.5%)	291 (100%)	1	0.300	298 (99.3%)	164 (99.4%)	1	0.770
		G/G	2 (0.5%)	0 (0%)	0.00 (0.00-NA)		2 (0.7%)	1 (0.6%)	0.59 (0.02-20.72)	
	Overdominant	A/A-G/G	321 (83.6%)	224 (77%)	1	0.041*	248 (82.7%)	137 (83%)	1	0.990
		G/A	63 (16.4%)	67 (23%)	**1.57 (1.02-2.43)**		52 (17.3%)	28 (17%)	0.99 (0.53-1.85)	
	Log-additive	---	---	---	1.47 (0.96-2.23)	0.074	---	---	0.97 (0.53-1.75)	0.910
rs12580300	Codominant	G/G	134 (35%)	98 (33.7%)	1	0.820	99 (33%)	63 (38.2%)	1	0.043*
		G/A	179 (46.7%)	147 (50.5%)	1.08 (0.73-1.59)		150 (50%)	82 (49.7%)	0.66 (0.39-1.13)	
		A/A	70 (18.3%)	46 (15.8%)	0.93 (0.56-1.55)		51 (17%)	20 (12.1%)	**0.39 (0.18-0.85)**	
	Dominant	G/G	134 (35%)	98 (33.7%)	1	0.840	99 (33%)	63 (38.2%)	1	0.039*
		G/A-A/A	249 (65%)	193 (66.3%)	1.04 (0.72-1.49)		201 (67%)	102 (61.8%)	**0.59 (0.35-0.97)**	
	Recessive	G/G-G/A	313 (81.7%)	245 (84.2%)	1	0.610	249 (83%)	145 (87.9%)	1	0.046
		A/A	70 (18.3%)	46 (15.8%)	0.89 (0.56-1.41)		51 (17%)	20 (12.1%)	0.50 (0.24-1.00)	
	Overdominant	G/G-A/A	204 (53.3%)	144 (49.5%)	1	0.570	150 (50%)	83 (50.3%)	1	0.590
		G/A	179 (46.7%)	147 (50.5%)	1.11 (0.78-1.56)		150 (50%)	82 (49.7%)	0.87 (0.54-1.42)	
	Log-additive	---	---	---	0.98 (0.77-1.26)	0.890	---	---	**0.63 (0.44-0.91)**	0.012*
rs7309325	Codominant	T/T	320 (83.5%)	226 (77.7%)	1	0.100	248 (82.7%)	137 (83.5%)	1	0.880
		G/T	62 (16.2%)	65 (22.3%)	1.55 (1.00-2.40)		52 (17.3%)	26 (15.8%)	1.02 (0.54-1.92)	
		G/G	1 (0.3%)	0 (0%)	0.00 (0.00-NA)		0 (0%)	1 (0.6%)	NA (0.00-NA)	
	Dominant	T/T	320 (83.5%)	226 (77.7%)	1	0.055	248 (82.7%)	137 (83.5%)	1	0.930
		G/T-G/G	63 (16.4%)	65 (22.3%)	1.53 (0.99-2.37)		52 (17.3%)	27 (16.5%)	1.03 (0.55-1.93)	
	Recessive	T/T-G/T	382 (99.7%)	291 (100%)	1	0.420	300 (100%)	163 (99.4%)	1	0.620
		G/G	1 (0.3%)	0 (0%)	0.00 (0.00-NA)		0 (0%)	1 (0.6%)	NA (0.00-NA)	
	Overdominant	T/T-G/G	321 (83.8%)	226 (77.7%)	1	0.047*	248 (82.7%)	138 (84.2%)	1	0.960
		G/T	62 (16.2%)	65 (22.3%)	**1.56 (1.01-2.41)**		52 (17.3%)	26 (15.8%)	1.02 (0.54-1.91)	
	Log-additive	---	---	---	1.49 (0.97-2.29)	0.066	---	---	1.04 (0.56-1.94)	0.900
		T/T	94 (24.4%)	78 (26.8%)	1		72 (24%)	44 (26.7%)	1	0.022*
	Codominant	C/T	196 (50.9%)	150 (51.5%)	0.80 (0.53-1.21)	0.480	152 (50.7%)	93 (56.4%)	0.82 (0.46-1.47)	
		C/C	95 (24.7%)	63 (21.6%)	0.76 (0.46-1.25)		76 (25.3%)	28 (17%)	**0.39 (0.19-0.81)**	
	Dominant	T/T	94 (24.4%)	78 (26.8%)	1	0.240	72 (24%)	44 (26.7%)	1	0.140
		C/T-C/C	291 (75.6%)	213 (73.2%)	0.79 (0.53-1.17)		228 (76%)	121 (73.3%)	0.66 (0.38-1.15)	
	Recessive	T/T-C/T	290 (75.3%)	228 (78.3%)	1	0.560	224 (74.7%)	137 (83%)	1	0.007*
rs2078863		C/C	95 (24.7%)	63 (21.6%)	0.89 (0.59-1.34)		76 (25.3%)	28 (17%)	**0.44 (0.24-0.82)**	
Overdominant	T/T-C/C	189 (49.1%)	141 (48.5%)	1		0.580	148 (49.3%)	72 (43.6%)	1	0.330
		C/T	196 (50.9%)	150 (51.5%)	0.91 (0.64-1.28)		152 (50.7%)	93 (56.4%)	1.27 (0.78-2.06)	
	Log-additive	---	---	---	0.87 (0.68-1.12)	0.270	---	---	**0.64 (0.44-0.91)**	0.012*
rs11879293		G/G	205 (53.5%)	176 (60.5%)	1	0.018*	160 (53.3%)	86 (52.1%)	1	0.760
	Codominant	G/A	148 (38.6%)	101 (34.7%)	**0.68 (0.47-0.99)**		125 (41.7%)	68 (41.2%)	0.83 (0.50-1.37)	
		A/A	30 (7.8%)	14 (4.8%)	**0.41 (0.19-0.89)**		15 (5%)	11 (6.7%)	0.93 (0.35-2.46)	
	Dominant	G/G	205 (53.5%)	176 (60.5%)	1	0.012*	160 (53.3%)	86 (52.1%)	1	0.490
		G/A-A/A	178 (46.5%)	115 (39.5%)	**0.64 (0.45-0.91)**		140 (46.7%)	79 (47.9%)	0.84 (0.52-1.37)	
Recessive	G/G-G/A	353 (92.2%)	277 (95.2%)	1	0.049*		285 (95%)	154 (93.3%)	1	0.990
		A/A	30 (7.8%)	14 (4.8%)	0.48 (0.23-1.02)		15 (5%)	11 (6.7%)	1.01 (0.39-2.60)	
	Overdominant	G/G-A/A	235 (61.4%)	190 (65.3%)	1	0.110	175 (58.3%)	97 (58.8%)	1	0.470
		G/A	148 (38.6%)	101 (34.7%)	0.75 (0.52-1.07)		125 (41.7%)	68 (41.2%)	0.84 (0.51-1.37)	
	Log-additive	---	---	---	**0.66 (0.50-0.89)**	0.005*	---	---	0.90 (0.61-1.32)	0.580
rs12232780	Codominant	G/G	215 (56.1%)	198 (68%)	1	0.002*				0.970
		G/A	146 (38.1%)	84 (28.9%)	**0.56 (0.39-0.82)**		191 (63.7%)	94 (57%)	1	
		A/A	22 (5.7%)	9 (3.1%)	**0.33 (0.13-0.84)**		102 (34%)	67 (40.6%)	1.03 (0.62-1.71)	
	Dominant	G/G	215 (56.1%)	198 (68%)	1	0.001*	7 (2.3%)	4 (2.4%)	0.86 (0.19-4.01)	0.930
		G/A-A/A	168 (43.9%)	93 (32%)	**0.53 (0.37-0.76)**		191 (63.7%)	94 (57%)	1	
	Recessive	G/G-G/A	361 (94.3%)	282 (96.9%)	1	0.047*	109 (36.3%)	71 (43%)	1.02 (0.62-1.68)	0.840
		A/A	22 (5.7%)	9 (3.1%)	0.41 (0.16-1.03)		293 (97.7%)	161 (97.6%)	1	
	Overdominant	G/G-A/A	237 (61.9%)	207 (71.1%)	1	0.007*	7 (2.3%)	4 (2.4%)	0.85 (0.19-3.92)	0.880
		G/A	146 (38.1%)	84 (28.9%)	**0.60 (0.42-0.88)**		198 (66%)	98 (59.4%)	1	
	Log-additive	---	---	---	**0.57 (0.41-0.78)**	0.000*	102 (34%)	67 (40.6%)	1.04 (0.63-1.72)	
	Codominant	C/C	188 (49.5%)	129 (44.3%)	1	0.013*	152 (50.7%)	78 (47.3%)	1	0.670
		T/C	171 (45%)	128 (44%)	1.05 (0.73-1.52)		130 (43.3%)	73 (44.2%)	1.15 (0.69-1.90)	
		T/T	21 (5.5%)	34 (11.7%)	**2.69 (1.36-5.32)**		18 (6%)	14 (8.5%)	1.47 (0.59-3.66)	
rs2072382	Dominant	C/C	188 (49.5%)	129 (44.3%)	1	0.270	152 (50.7%)	78 (47.3%)	1	0.470
		T/C-T/T	192 (50.5%)	162 (55.7%)	1.21 (0.86-1.72)		148 (49.3%)	87 (52.7%)	1.20 (0.74-1.94)	
	Recessive	C/C-T/C	359 (94.5%)	257 (88.3%)	1	0.003	282 (94%)	151 (91.5%)	1	0.470
		T/T	21 (5.5%)	34 (11.7%)	2.62 (1.36-5.06)		18 (6%)	14 (8.5%)	1.38 (0.57-3.32)	
	Overdominant	C/C-T/T	209 (55%)	163 (56%)	1	0.620	170 (56.7%)	92 (55.8%)	1	0.740
		T/C	171 (45%)	128 (44%)	0.91 (0.65-1.30)		130 (43.3%)	73 (44.2%)	1.09 (0.67-1.77)	
	Log-additive	---	---	---	1.35 (1.02-1.77)	0.034	---	---	1.19 (0.81-1.73)	0.380

OR = odds ratio; 95 % CI = 95 % confidence interval

*p* values were calculated by unconditional logistic regression adjusted for age

*p < 0.05 indicates statistical significance

**Figure 1:**
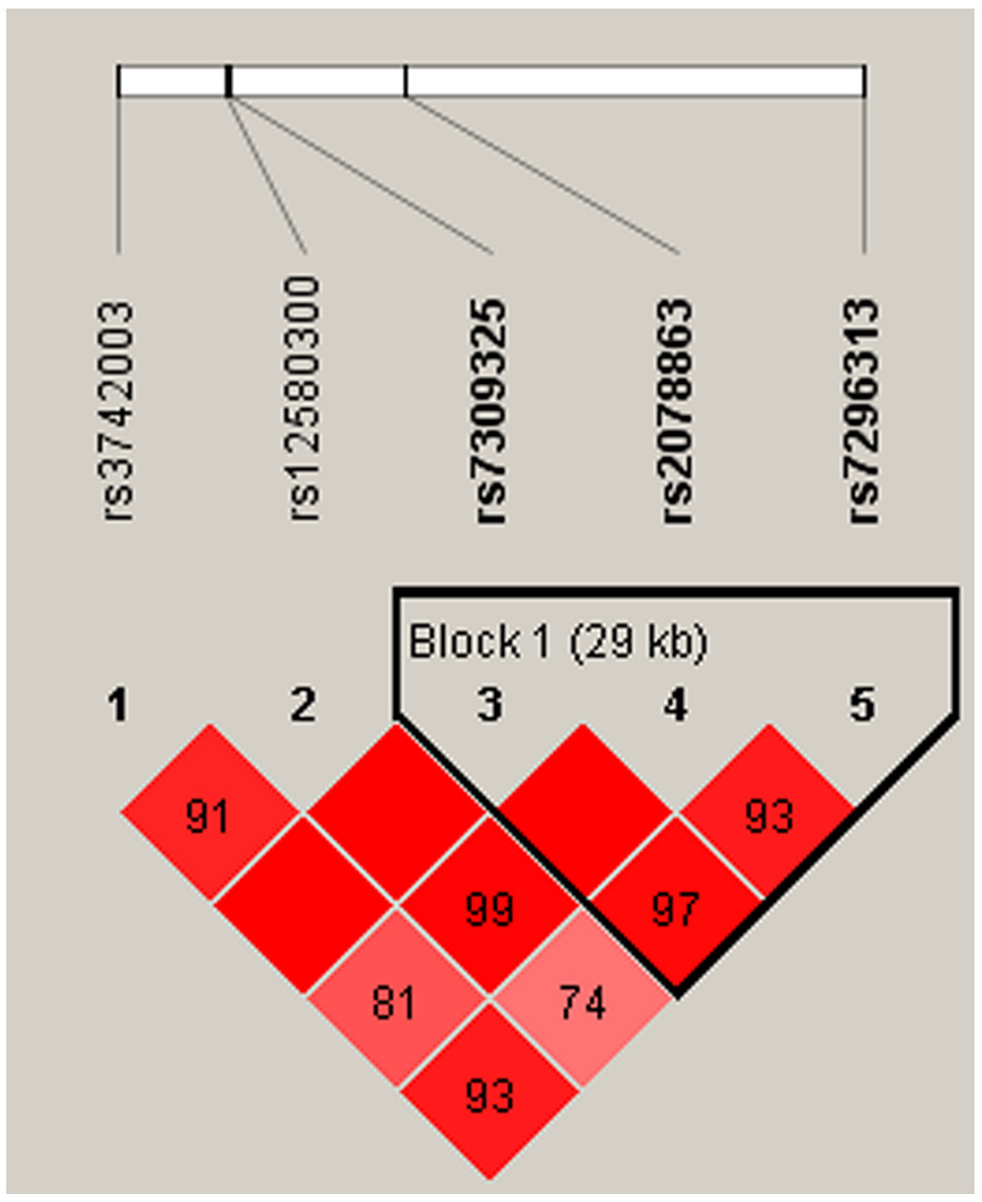
Haplotype block map for SNPs of the *SH2B3* gene in the women population

**Figure 2:**
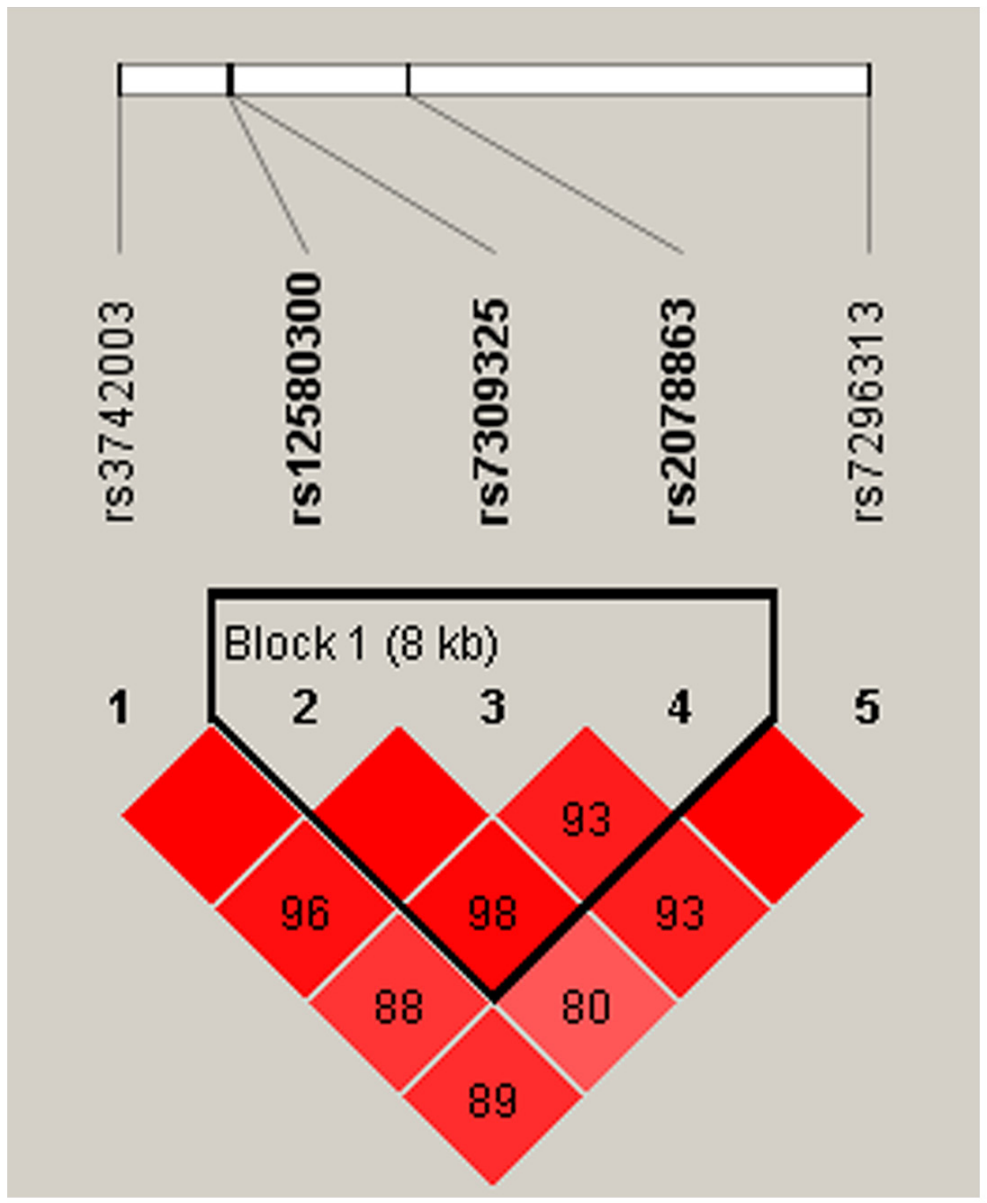
Haplotype block map for SNPs of the *SH2B3* gene in the men population

We also observed that some haplotypes of the *SH2B3* gene were associated with CAD risk. However, the specific haplotypes involved were different in the men and women. One block (rs7309325, rs2078863, rs7296313) showed to be associated with CAD risk only in women (Figure 1, Table 4). This association resulted from a modest but significant decrease in the frequency of the “TTC” haplotype in female patients compared with controls (OR = 0.59, 95% CI: 0.40-0.86; *p* = 0.007). Meanwhile, the haplotype “GTT” in another block (rs12580300, rs7309325, rs2078863) was associated with increased susceptibility to CAD (OR = 1.59, 95% CI: 1.01-2.50; *p* = 0.047) in the men population (Table 5, Figure 2). All of the above results were adjusted by age.

**Table 4:** *SH2B3* haplotype frequencies and CAD risk association in the women group

Gene	SNP	Haplotype	Frequency (%)		OR 95%CI	*p*
			Cases	Controls		
SH2B3	rs7309325|rs2078863|rs7296313	TCC	0.545	0.492	1	---
		TTC	0.361	0.451	0.59 (0.40 - 0.86)	0.007*
		GTT	0.082	0.085	0.79 (0.41 - 1.53)	0.490
		---	---	---	0.97 (0.18 - 5.15)	0.970

OR = odds ratio; 95 % CI = 95 % confidence interval

*p* values were calculated using two-sided Chi-square test without adjustment for age

*p < 0.05 indicates statistical significance

**Table 5:** *SH2B3* haplotype frequencies and CAD risk association in the men group

Gene	SNP	Haplotype	Frequency (%)		OR 95%CI	*p*
			Cases	Controls		
SH2B3	rs12580300|rs7309325|rs2078863	GTC	0.471	0.495	1	---
		ATT	0.41	0.413	1.07 (0.82 - 1.39)	0.61
		GTT	0.108	0.081	1.59 (1.01 - 2.50)	0.047*
		---	---	---	0.82 (0.24 - 2.79)	0.75

OR = odds ratio; 95 % CI = 95 % confidence interval

*p* value from were calculated from two-sided Chi-squared test without adjusted by age

*p < 0.05 indicates statistical significance

Next, *SH2B3* and *SMARCA4* gene polymorphisms were analyzed to establish their potential associations with study subjects’ lipid profiles, including triglyceride (TG), total cholesterol (TC), apolipoprotein A1/apolipoprotein B (APOA1/APOB), and HDL and LDL cholesterol (Table 6). Results showed that LDL cholesterol levels in rs3742003 G allele carriers were higher in cases versus controls in the male population (*p* = 0.049). In addition, LDL cholesterol levels were associated with rs7309325 (*p* = 0.048), rs7296313 (*p* = 0.040), and rs11879293 (*p* = 0.013) in the male population. In the female group, in contrast, we detected associations between rs3742003, rs7309325 and rs7296313, and APOA1/APOB levels. There were no significant differences between cases and controls in the other lipids profiled (Table 6).

**Table 6:** Lipid levels measured for SNP genotypes in the men and women populations

SNP	TG (mmol/L)	*p*	TC (mmol/L)	*p*	HDL (mmol/L)	*p*	LDL (mmol/L)	*p*	APOA1/APOB	*p*
**Men population (n = 291)**										
rs3742003										
A/A (n = 221)	1.8687 ± 1.798	0.211	3.929 ± 1.14474	0.455	1.0817 ± 0.247	0.055	1.8005 ± 0.682	0.049*	1.438 ± 0.744	0.726
G/A (n = 64)	1.5745 ± 0.962		4.0567 ± 1.321		1.1503 ± 0.257		2.0514 ± 1.364		1.473 ± 0.609	
rs12580300										
G/G (n = 92)	1.6597 ± 1.1596	0.587	3.99 ± 1.193	0.610	1.108 ± 0.264	0.768	2.0148 ± 1.163	0.107	1.487 ± 0.988	0.463
A/A (n = 41)	1.8168 ± 1.3119		4.0898 ± 1.038		1.1102 ± 0.273		1.8382 ± 0.597		1.327 ± 0.480	
G/A (n = 139)	1.8881 ± 1.9825		3.8954 ± 1.2286		1.0868 ± 0.234		1.7623 ± 0.742		1.457 ± 0.542	
rs7309325										
T/T (n = 213)	1.8592 ± 1.7928	0.271	3.9294 ± 1.1429	0.451	1.0824 ± 0.2458	0.062	1.8017 ± 0.6819	0.048*	1.437 ± 0.7411	0.709
G/T (n = 62)	1.5976 ± 0.9665		4.0595 ± 1.3322		1.1498 ± 0.2611		2.0555 ± 1.3812		1.476 ± 0.6161	
rs7296313										
C/C (n = 215)	1.8807 ± 1.7968	0.125	3.9401 ± 1.1415	0.628	1.0864 ± 0.2548	0.158	1.8005 ± 0.6803	0.040*	1.442 ± 0.7444	0.864
T/C (n = 60)	1.5122 ± 0.8676		4.0247 ± 1.3458		1.138 ± 0.2319		2.0682 ± 1.400		1.46 ± 0.0772	
rs11879293										
G/G (n = 172)	1.933 ± 1.9368	0.224	3.8893 ± 1.0763	0.140	1.0934 ± 0.2457	0.324	1.7395 ± 0.6508	0.013*	1.485 ± 0.7881	0.511
A/A (n = 13)	1.6231 ± 0.9815		3.6023 ± 1.2987		1.01 ± 0.1292		1.9146 ± 0.7138		1.369 ± 0.5040	
G/A (n = 90)	1.5721 ± 0.9515		4.1438 ± 1.3499		1.1184 ± 0.2708		2.079 ± 1.2209		1.383 ± 0.5787	
rs2072382										
C/C (n = 119)	1.745 ± 1.1847	0.573	3.9791 ± 1.2366	0.081	1.0771 ± 0.2341	0.257	1.8449 ± 0.8019	0.081	1.457 ± 0.6025	0.021*
T/C (n = 122)	1.9077 ± 1.9746		4.0543 ± 1.1807		1.1254 ± 0.2679		1.9537 ± 1.0251		1.354 ± 0.5	
T/T (n = 34)	1.6082 ± 1.7521		3.5421 ± 0.9466		1.0697 ± 0.2378		1.5679 ± 0.5779		1.735 ± 1.3715	
**Women population (n = 165)**										
rs3742003										
A/A (n = 127)	1.6872 ± 0.7916	0.069	4.3562 ± 1.0572	0.304	1.2002 ± 0.2547	0.550	2.0403 ± 0.6762	0.085	1.405 ± 0.5733	0.036*
G/A (n = 27)	2.3096 ± 2.5019		4.153 ± 1.0764		1.1448 ± 0.3397		1.8841 ± 0.8282		1.785 ± 1.1370	
rs12580300										
G/G (n = 62)	1.9269 ± 1.7576	0.574	4.2015 ± 0.8815	0.464	1.1535 ± 0.2472	0.080	1.9276 ± 0.6519	0.368	1.434 ± 0.6677	0.285
A/A (n = 19)	1.6637 ± 0.7636		4.3642 ± 1.2542		1.1268 ± 0.2492		2.1495 ± 0.7979		1.274 ± 0.5184	
G/A (n = 73)	1.72 ± 0.8241		4.4273 ± 1.1479		1.2365 ± 0.2505		2.0682 ± 0.7359		1.551 ± 0.7881	
rs7309325										
T/T (n = 127)	1.6842 ± 0.7911	0.060	4.3565 ± 1.0572	0.305	1.2004 ± 0.2546	0.560	2.0382 ± 0.6769	0.094	1.408 ± 0.5727	0.036*
G/T (n = 26)	2.3358 ± 2.5476		4.1492 ± 1.0976		1.145 ± 0.2445		1.8919 ± 0.8436		1.796 ± 1.1581	
rs7296313										
C/C (n = 127)	1.694 ± 0.7912	0.097	4.3457 ± 1.0468	0.373	1.1967 ± 0.2546	0.753	2.032 ± 0.6697	0.113	1.402 ± 0.511	0.029*
T/C (n = 27)	2.2774 ± 2.5105		4.2022 ± 1.1328		1.1611 ± 0.2437		1.9233 ± 0.8599		1.796 ± 0.218	
rs11879293										
G/G (n = 83)	1.8205 ± 1.5942	0.929	4.1941 ± 0.9307	0.221	1.1711 ± 0.2454	0.550	1.9389 ± 0.6324	0.051	1.473 ± 0.6894	0.323
A/A (n = 11)	1.6664 ± 0.8972		4.3755 ± 1.1683		1.2436 ± 0.3385		1.75 ± 0.6879		1.764 ± 1.1986	
G/A (n = 61)	1.7857 ± 0.7488		4.5048 ± 1.1942		1.2057 ± 0.2444		2.1839 ± 0.7899		1.411 ± 0.6327	
rs2072382										
C/C (n = 74)	1.9289 ± 1.5378	0.358	4.3726 ± 0.9984	0.892	1.1814 ± 0.2459	0.303	1.9684 ± 0.6406	0.609	1.432 ± 0.6319	0.803
T/C (n = 70)	1.7254 ± 1.0477		4.2901 ± 1.1109		1.2163 ± 0.2589		2.0529 ± 0.7795		1.496 ± 0.6449	
T/T (n = 13)	1.4443 ± 0.4935		4.2929 ± 1.2073		1.1057 ± 0.2394		2.1507 ± 0.7514		1.543 ± 1.2924	

Triglyceride = TG; Total Cholesterol = TC; Apolipoprotein A1/ Apolipoprotein B = APOA1/APOB;

High-density lipoprotein = HDL; Low-density lipoprotein = LDL

*p* values were calculated using *t* test

*p < 0.05 indicates statistical significance

## DISCUSSION

In the present case-control study we confirmed striking sex-related differences in the association of polymorphisms of the *SH2B3* and *SMARCA4* genes with CAD risk. Thus, certain *SH2B3* SNPs, i, e, rs12580300 and rs2078863, were associated with CAD risk in women. In addition, we also underscored associations between *SMARCA4* gene SNPs and CAD risk in men. These observations may explain differences in the propensity for early CAD development in men and women and may have potential sex-specific therapeutic implications.

The *SH2B3* gene encodes SH2B adaptor protein 3, a member of the Src homology 2-B (SH2B) adaptor family. For a long time, the functions of the *SH2B3* gene remained poorly understood, until it was shown to affect several traits linked to CAD, including regulation of hematopoiesis and cytokine signaling. A recent study demonstrated that a *SH2B3* polymorphism was associated with both lower LDL cholesterol and HDL cholesterol concentration [25], whereas another study pointed the association of CAD risk loci in the *SH2B3* gene with regulation of blood pressure [10].

Two SNPs in the *SH2B3* gene showed an association with CAD risk in our study; one was associated with decreased risk in women and the other with increased risk. rs12580300, an intron-variant in the *SH2B3* gene, was found to be associated with decreased CAD risk in the Chinese population for the first time. However, such association is not entirely clear. In contrast, rs2078863, also located in an intronic region of the *SH2B3* gene, increased CAD risk and showed the strongest, so far unreported, association signal with CAD for *SH2B3*. Both loci were selected randomly. Therefore, further studies should be performed to verify the association.

*SMARCA4* encodes a protein that belongs to the SWI/SNF protein family. It is the central catalytic component of the SWI/SNF complex, which involves multiple domains including an evolutionarily conserved catalytic ATPase domain, a conserved C-terminal bromodomain, an AT-hook motif, and the less characterized N-terminal region, all of which participate in the recognition of modified histone proteins, DNA binding, or recruitment of SWI/SNF [26, 27]. *SMARCA4* is involved in various cellular processes including transcriptional regulation, cell cycle control, proliferation, DNA repair, and recombination [28]. Recently, the association of *SMARCA4* with CAD risk has been highlighted by GWAS and other methods [22, 24, 29, 30]. Accordingly, our analysis shows that *SMARCA4* rs11879293 and rs12232780 are associated with reduced risk of CAD in men, a result consistent with previous research by Martinelli and colleagues [23].

Our study is the first to demonstrate that CAD pathogenesis is influenced by sex differences in polymorphisms in *SMARCA4* and *SH2B3* genes. Although this study had sufficient statistical power, there were still some intrinsic limitations. First, we investigated only 5 genetic variants in each gene, and cannot exclude the possibility that other polymorphisms might also be associated with CAD risk. Also, we did not found an association between rs1122608 in *SMARCA4* and CAD risk, as suggested by previous research [23]. Second, the association between *SMARCA4* and *SH2B3* polymorphisms and clinical information was not evaluated in this study. In addition, no significant associations were observed between the SNPs and the risk of CAD after Bonferroni correction. This may be due to our relatively small sample size, the SNP selection criteria (minor allele frequency > 5%), and inherent weaknesses of the Bonferroni correction itself (the interpretation of the results depends upon the number of comparisons performed). Multiple independent studies with large sample sizes are required to validate our findings.

## MATERIALS AND METHODS

### Subjects

The study included 456 Chinese CAD patients (291 men and 165 women) and 685 controls (385 men and 300 women) enrolled from September 2014 to October 2015 at the First hospital of Xi’an, China. The subjects presented no within-group relatedness. The Judkins approach [31] was used by highly skilled physicians to perform all coronary angiography procedures. A cardiologist diagnosed each patient according to applicable diagnostic criteria such as typical ischemic discomfort, electrocardiographic changes, increases in cardiac markers including creatine kinase-MB and troponin T, and coronary angiography outcomes (coronary diameter ≥ 2 mm; stenosis ≥ 50%). At least two experienced imaging specialists interpreted the coronary angiography findings, and the final CAD diagnosis was made based on the angiography report. Exclusion criteria included additional syndromes, multiple developmental abnormalities, or known chromosomal abnormalities. CAD patients were also excluded if their mothers had maternal diabetes mellitus, phenylketonuria, and teratogen or therapeutic drug exposure during pregnancy. In addition, 685 age-matched Chinese control subjects (ages 50–80 years) without signs or symptoms of CAD were recruited from the same hospital over the same time period. All control participants underwent a coronary angiogram confirming absence of coronary artery stenoses, and showed no clinical or electrocardiographic evidence of myocardial infarction or CAD. We recorded detailed information about the participants, including their personal medical history (hypertension, diabetes mellitus, etc.), familial medical history, reproductive history, menopausal status, and lifestyle habits (smoking, drinking, etc.). All subjects underwent a clinical examination at which a blood sample was drawn for routine blood analysis, biochemical tests, coagulation function, and genetic analyses. Informed consents were obtained from all participants. This study was approved by the ethical committee of Xi’an Jiaotong University, School of Medicine, Xi’an, China.

### SNP selection and genotyping

This study selected two CAD-susceptibility genes, *SH2B3* and *SMARCA4*, identified in previous research [25, 32]. SNPs within these genes were selected randomly from the HapMap database. Ten candidate SNPs (rs3742003, rs12580300, rs7309325, rs2078863, rs7296313, rs11879293, rs12232780, rs2072382, rs1529729, and rs1122608) in the *SH2B3* and *SMARCA4* genes with minor allele frequencies (MAF) >5 % in Asians were finally selected for genotyping.

We collected 5 ml venous blood samples in EDTA-containing tubes and stored them at -20˚C. DNA was obtained from whole blood leukocytes using the Gold Mag-Mini Whole Blood Genomic DNA Purification Kit (version 3.0; TaKaRa, Japan) [33] and its purity and concentration were determined spectrophotometrically by measuring the absorbance at 260 and 280 nm. Finally, genotypes derived from pure, integrated, and high-quality DNA samples were analyzed.

Genotyping was conducted using a Sequenom MassARRAY RS1000 (Sequenom, Inc.) following the manufacturer's instructions. Briefly, it included a polymerase chain reaction (PCR) amplification assay, designing of primers and probes, purification with Shrimp Alkaline Phosphatase (SAP), addition of primers and extension of the basic group, followed by stimulating co-crystallization by combining sample analyte and chip substrates. The primers were designed using Sequenom MassARRAY Assay Design 3.0 software [34]. The overall success rate of all the genotyping assays was over 98%.

### Statistical methods

All statistical analyses were performed using SPSS version 17.0 for Windows (SPSS, Chicago, IL). Differences in categorical and continuous variables between cases and controls were tested using the chi-square (χ^2^) test and the Student's t-test, respectively. χ^2^ test for genotype distribution was conducted to evaluate deviation from Hardy–Weinberg equilibrium (HWE) for the ten SNPs. Genotype and allele distributions for SNPs between controls and CAD groups were also compared by χ^2^ test. All of the minor alleles were regarded as risk alleles for CAD susceptibility. Unconditional logistic regression, with adjustment for age, was used to estimate relative risk of CAD for each of the tested genotypes in the form of odds ratio (OR) and 95 % confidence interval (CI). All statistical tests were 2-sided and a *p* < 0.05 was considered as statistically significant. Linkage disequilibrium analysis and SNP haplotypes were done using the Haploview software package (version 4.2) and the SHEsis software platform (http://analysis.bio-x.cn/myAnalysis.php).

## CONCLUSION

This is the first study examining shared genetic influences on CAD risk and revealing a strong sex-dependence for these associations. With basis on these findings, further studies on the contribution of germline genetic variants in the *SH2B3* and *SMARCA4* genes to CAD risk are warranted.
